# “Vascular” Korsakoff Syndrome With Bilaterally Damaged Mammillothalamic Tracts: Insights Into the Pathogenesis of “Acute” Korsakoff Syndrome As Acute-Onset Irreversible Anterograde Amnesia

**DOI:** 10.7759/cureus.19472

**Published:** 2021-11-11

**Authors:** Yuichiro Yoneoka, Yasuhiro Seki, Katsuhiko Akiyama

**Affiliations:** 1 Department of Neurosurgery, Uonuma Kikan Hospital, Uonuma Institute of Community Medicine, Niigata University Medical and Dental Hospital, Minami-Uonuma, JPN

**Keywords:** vascular korsakoff syndrome, irreversible anterograde amnesia, anterograde amnesia, magnetic resonance imaging, pathogenesis of korsakoff syndrome, microbleeds, acute cerebral infarction, papez circuit, mammillothalamic tract, non-alcoholic korsakoff syndrome

## Abstract

The structural pathogenesis of Wernicke-Korsakoff syndrome remains debatable. Wernicke encephalopathy is acute and often reversible whereas Korsakoff syndrome (KS) is chronic and may be irreversible. The cognitive deficits observed in KS are considered to be primarily due to damage to the anterior nucleus of the thalamus, mammillary bodies, and corpus callosum. We present an extremely rare case of non-alcoholic “vascular” KS (vKS) as acute-onset amnesia. A 97-year-old man living alone was brought to our hospital, complaining of sudden-onset behavioral changes with amnesia. Diffusion-weighted images (DWIs) showed fresh cerebral infarction in the right thalamus involving the right mammillothalamic tract (MTT). T2*-weighted images (T2*WIs), in addition, revealed a microbleed scar over the left MTT. This case supports the hypothesis that bilateral MTT dysfunction can lead to KS. Furthermore, in collaboration with a prior report about non-alcoholic “acute” KS due to cerebral infarction, this case supports the existence of vascular KS as an acute-onset amnestic syndrome, as well as insight into the pathogenesis of KS as an irreversible amnestic syndrome.

## Introduction

Korsakoff syndrome (KS) is a chronic memory disorder caused by a severe deficiency of thiamine most commonly observed in alcoholics. However, some have proposed that focal structural lesions disrupting memory circuits, in particular, the mammillary bodies, the mammillothalamic tract (MTT), and the anterior thalamus, can give rise to this amnestic syndrome [1]. Here, the authors present an elderly patient with sudden-onset irreversible KS caused by right thalamic infarction involving the right MTT in collusion with a left thalamic hemorrhagic scar over the left MTT. Furthermore, in collaboration with a prior report about non-alcoholic “acute” KS due to unilateral thalamic infarction [2], this case proves the existence of “vascular” KS (vKS) as an acute-onset amnestic syndrome, as well as insight into the pathogenesis of KS as an irreversible amnestic syndrome.

## Case presentation

**Figure 1:**
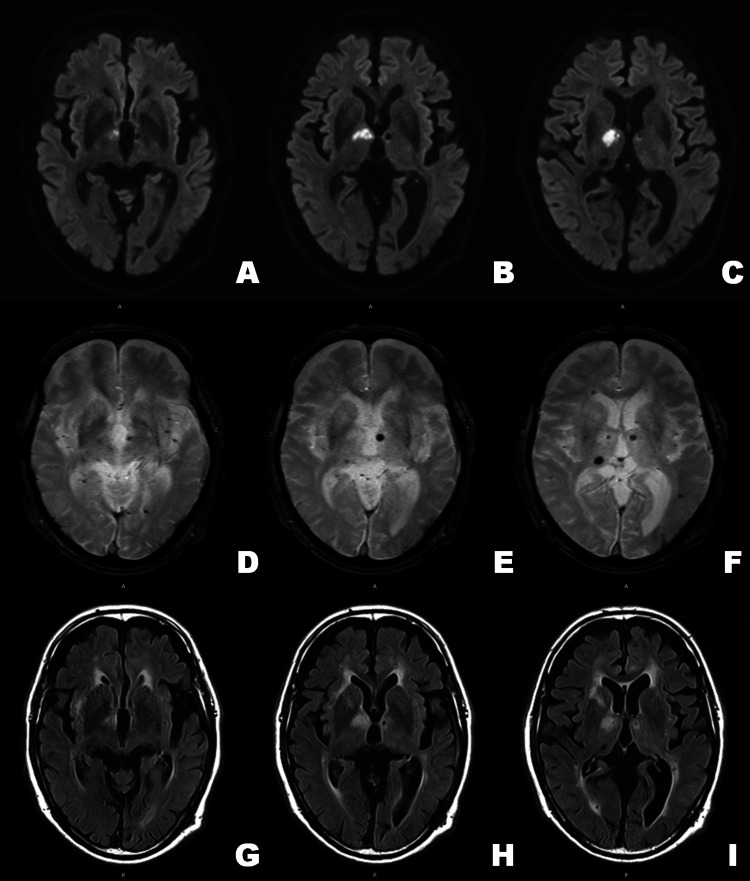
Bilateral damage of the mammillothalamic tracts. MRIs on admission. Diffusion-weighted images show the acute infarction over the right mammillothalamic tract (MTT) (A-C). T2* weighted images reveal a microbleed over the left MTT (D-F). Fluid attenuation inversion recovery images confirm bilateral MTTs and his age-appropriate brain except for both of the MTTs. Note the dilated left trigone of the left lateral ventricle, indicating the left MTT damage is old.

A 97-year-old man living alone complained of sudden-onset behavioral changes with amnesia lasting two days. He was brought to our hospital by his son. The patient had been well until the day of onset, living alone by himself. The day before the first visit (Day 01), his neighbors saw him murmuring repeatedly about not understanding the reason for the current action. The son was told about his father’s behavioral change of confabulation by one of the neighbors, and then the son brought his father to the ER on the next day (Day 02). On examination, the patient was awake and oriented about his distant memories, however, he would express minimal content in conversation, repeating the same questions several times during his stay in the ER. His neurological examination showed normal eye movements without ophthalmoplegia and without paresis or ataxia of the extremities. He could walk independently in an age-appropriate manner as usual. No traumatic findings were found on his body. He remembered the Great East Japan Earthquake and its associated Tsunamis in 2011 but not the Niigata-Chuetsu Earthquake (Mid-Niigata Prefecture earthquake) in 2004 nor the Niigata Chuetsu-offshore Earthquake in 2007. He did not remember his wife’s death in 2009. His blood levels of glucose, calcium, phosphorus, lipase and amylase were normal, as were the results of liver and renal function tests. Diffusion-weighted images (DWIs) showed fresh cerebral infarction in the right thalamus involving the right MTT (Figures 1A-1C).

**Figure 2:**
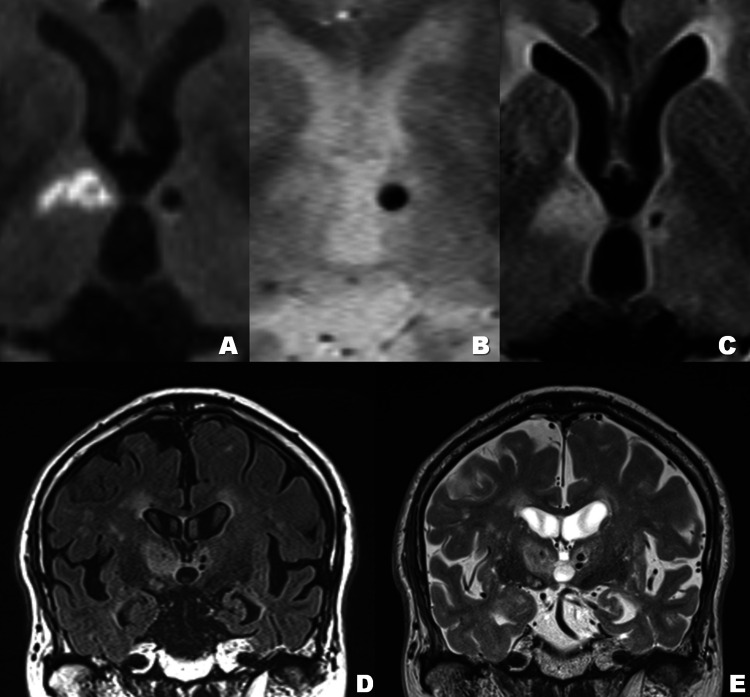
Closed view of the bilateral mammillothalamic tracts. Enlarged views of MRIs demonstrate infarction over the right mammillothalamic tracts (MTTs) (A) and a microbleed over the left MTT (B). Fluid attenuation inversion recovery shows the right fresh MTT ischemic lesion and the left old MTT lesion (C). Coronal images confirm bilateral symmetrical locations of the MMT lesions from (D-E). The right MTT infarction localizes between the right mammillary body and the right anterior thalamic nucleus (D-E). The left MTT microbleeds localize between the left mammillary body and the right anterior thalamic nucleus (D-E).

Additionally, T2*-weighted images (T2*WIs) revealed a microbleed scar over the left MTT (Figures 1D-1F). Fluid attenuation inversion recovery images confirmed bilateral lesions on both the MTTs (Figures 1G-1I). Closed image reading revealed infarction over the right MMT and microbleed over the left MMT (Figures 2A-2E).

**Figure 3:**
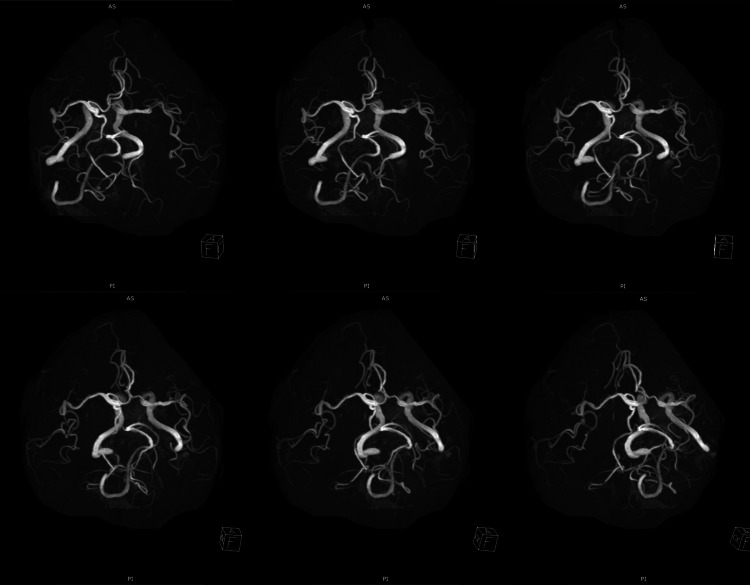
Magnetic resonance angiography. Magnetic resonance angiography shows an age-appropriate arterial system. Major arteries are patent. No vascular anomalies are detected.

Magnetic resonance (MR) angiography showed an age-appropriate arterial system (Figure 3).

We diagnosed this ischemic lesion as lacunar infarction. The patient was started on antiplatelet therapy with peroral aspirin after admission since the risk of ischemic stroke was higher than that of cerebral hemorrhage, regardless of cerebral microbleeds presence, for the patient. He was rehabilitated to maintain his strength and to regain the ability necessary to live by himself once again. Follow-up MRIs on Day 07 showed bilaterally damaged MTTs (Figures 2D-2E) without newly identified lesions. During his hospitalization for four weeks, his anterograde amnesia with some retrograde amnesia continued without improvement. After his hospitalization for acute medical care ended, he transferred to a rehabilitation facility for the elderly. His amnesia has continued.

## Discussion

The rarity of vascular Korsakoff syndrome with bilateral mammillothalamic tracts

The structural pathogenesis of Wernicke-Korsakoff syndrome remains debatable. Wernicke encephalopathy is acute and often reversible while KS is chronic and may be irreversible [3, 4]. KS is a chronic neuropsychiatric disorder that is caused by thiamine deficiency. In the industrialized world, the most common cause of thiamine deficiency is alcoholism, with around 90% of the deficiencies associated with alcohol abuse [5-7]. Around 15% of chronic alcoholics have neurological signs of KS [8]. The most prominent symptom of KS is a profound declarative memory impairment for learning and remembering new material (anterograde amnesia). In KS, there are also temporally graded memory deficits for remote memory (retrograde amnesia) which characteristically extend back many years or decades [9]. The cognitive problems in KS are caused by diencephalic atrophy of the brain, with damage to the anterior nucleus of the thalamus, the mammillary bodies, and the corpus callosum as the most common features of KS that are not caused by the direct neurotoxic effects of alcohol [8, 10-14].

Non-alcoholic “acute” Korsakoff syndrome

Previously, we reported a case of non-alcoholic “vascular” amnestic syndrome as “acute” KS [2]. In addition to the previous report [2], this report also describes a case of non-alcoholic “vascular” amnestic syndrome. In both cases, MRI demonstrates bilaterally damaged MTTs by infarction or microbleed. Although Savastano LE et al. reported rapid reversal KS caused by retrochiasmatic suprasellar tumors [1], the amnestic syndrome is irreversible in both of our cases. Also, in both of our cases, there is a common onset form. Based on unilateral damaged MTT caused by cerebrovascular disease, a newly added lesion involving the contralateral MTT results in sudden-onset permanent amnestic syndrome: acute irreversible KS, more practically vKS.

Existence of compensating mechanism of the Papez circuit

The MTT connects the mammillary body and the anterior thalamus. As a part of the Papez circuit, it is involved in episodic memory [15]. Within the Papez circuit, the mammillary bodies are considered to be relay nuclei, passing information from the hippocampal formation to the anterior thalamic nuclei, by way of the MTT [16, 17]. All neurons within the mammillary bodies are thought to project to the anterior thalamic nuclei consistent with the mammillary bodies acting as a relay [16, 17]. Based on these findings, we can conclude that, in unilateral MTT damage, the non-damaged side can be compensated for signal transmissions in the Papez circuit and does not manifest amnesia. In our case, compared with the right hippocampus, the coronal images reveal left hippocampal atrophy, indicating the left MTT lesion is an old lesion and may have caused this left hippocampal atrophy (Figures 2D-2E). This fact shows that unilateral MTT damage, as well as unilateral hippocampal atrophy, does not necessarily cause amnesia. Three other reports supporting this point are available [18-20].

Order of the side of MTT damage

Our previously reported case [2] has the damaged MTT on the left, and this case has the damaged MTT on the right. Albeit only two cases, the order of the side of MTT damage does not matter for the establishment of vKS. At this point, there is no clear functional dominancy between the right and left MTTs.

Views to be drawn by vascular Korsakoff syndrome with bilateral MTT damage

To summarize, the two cases of vKS reported by us (the present case and the previously reported case in reference 2) indicate the following seven points: 1) bilateral cerebrovascular MTT damage causes vKS, 2) vKS is acute onset, 3) unilateral MTT damage does not necessarily cause amnesia, 4) vKS is irreversible, 5) order of the side of MTT damage does not matter for the establishment of vKS, 6) there is no clear functional dominancy between both MTTs, and 7) unilateral MTT damage may cause ipsilateral hippocampal atrophy. These points provided by our two cases (the present case, 2] and similar reports [18-20] are powerful clues to investigate the pathogenesis of KS relating human Papez circuit, not limited to vKS.

## Conclusions

Under unilateral MTT damage caused by cerebrovascular disease, a newly added lesion involving the contralateral MTT results in sudden-onset permanent amnestic syndrome: vKS. Unilateral MTT damage alone will not necessarily cause amnesia. Order of the side of MTT damage does not matter for the establishment of vKS, indicating that there is no clear functional dominancy between the right and left MTTs. Unilateral MTT damage will cause ipsilateral hippocampal atrophy. These novel findings from cases of vKS will promote future research of the pathogenesis of KS, not just vKS. More precise recognition and more accumulation of vKS are warranted to investigate the pathogenesis of vKS, as well as of KS, consequently the mechanism of the human Papez circuit.
